# Energy- and Cost-Efficient Healthcare Task Offloading via Network Edge Digital Twin

**DOI:** 10.3390/s26154768

**Published:** 2026-07-27

**Authors:** Ayesha Jadoon, Hao Ran Chi, Daniel Corujo, Francisco J. Ferrão, Rui L. Aguiar

**Affiliations:** 1 Instituto de Telecomunicações, University of Aveiro, 3810-193 Aveiro, Portugal; ayeshajadoon@ua.pt (A.J.); dcorujo@av.it.pt (D.C.); ruilaa@ua.pt (R.L.A.); 2 Data Machine Elite, 2580-654 Lisbon, Portugal; francisco@f-ferrao.com

**Keywords:** digital twin, edge computing, healthcare IoT, resource fairness, energy consumption, DT freshness, heuristic approaches

## Abstract

This paper proposes a digital twin (DT)-enabled edge computing framework for healthcare task offloading in smart medical environments. The DT maintains a continuously synchronized virtual representation of the physical edge system, capturing queue states, resource utilization, and energy dynamics. Based on this virtual state, a deterministic optimization model is formulated to support real-time offloading and scheduling decisions under latency, energy, and fairness constraints. Unlike prediction-only approaches, the proposed DT operates in a closed-loop manner, where state estimation and synchronization directly influence scheduling feasibility and system performance. The offloading problem is formulated as a mixed-integer linear programming (MILP) model to jointly optimize task allocation, delay minimization, and energy efficiency. The simulated workload includes 10,000 heterogeneous healthcare applications. At each time slot, one to five tasks are generated and uniformly selected from ECG, video-processing, or medical-imaging workloads, with average task sizes of 90 KB, 280 KB, and 150 KB, respectively. This setup aims to emulate diverse real-world healthcare edge workloads with varying communication and computation demands. The proposed approach significantly reduces average latency by up to 57%, eliminates task drops in all evaluated scenarios, and improves load balancing compared with hospital-only and round-robin baselines. Although total energy consumption increases moderately, energy efficiency per completed task improves due to more effective scheduling by stability, reducing deadline violations and enhancing resource utilization. We further analyze the impact of DT freshness and updated frequency and show that outdated or misaligned DT updates can degrade performance by increasing delay and leading to suboptimal decisions, while overly frequent updates introduce additional coordination overhead. These results highlight the importance of jointly designing DT synchronization mechanisms and optimization-based scheduling strategies for reliable and cost-efficient healthcare edge systems.

## 1. Introduction

The Internet of Things (IoT) has become an important part of modern healthcare systems by enabling continuous and real-time monitoring through connected wearable sensors, medical devices, and edge computing technologies [1,2]. These IoT-based systems enable efficient collection, transmission, and processing of patient data, improving healthcare services through early disease detection, personalised treatments, and remote patient monitoring [3]. However, the growing number of connected IoT devices also creates major challenges in managing and scheduling computational tasks. In reference [4], healthcare IoT environments often face limited computing and energy resources, changing workloads, strict response-time requirements, and the need for reliable data processing in critical medical situations. Because of these factors, efficiently distributing and scheduling tasks across IoT networks is a difficult, complex problem.

To overcome these challenges, DT technology provides a promising solution. A DT acts as a virtual model of physical IoT devices and systems, allowing real-time monitoring, predictive analysis, and making smarter decisions [5]. When DT-based frameworks are integrated into healthcare systems, task scheduling and resource management can be significantly optimized. The results are shorter delays, better use of resources, improved energy efficiency, and overall more reliable healthcare services.

Despite these advantages, ref. [6] evidences that healthcare IoT systems generate large amounts of diverse, time-sensitive data that need to be processed in real time. Traditional cloud-centric architectures often fail to satisfy the strict latency and reliability requirements of such environments due to transmission delays, network congestion, and the overhead associated with transferring large data volumes [7]. These limitations are particularly critical in delay-sensitive applications such as electrocardiogram (ECG) analysis, medical image processing, and real-time patient monitoring, where even modest delays can affect service quality and operational responsiveness.

An effective edge computing solution that moves computational resources closer to data sources [8]. Compared with centralised cloud computing in reference [9], edge computing reduces communication latency, alleviates traffic on the backbone network, and improves privacy by keeping sensitive medical data within hospital boundaries. In DT-assisted healthcare networks, edge servers deployed near hospital wards, imaging laboratories, or IoT gateways can collaboratively process computationally intensive workloads in near-real time. In particular, as shown in reference [10], edge computing enables complex processing to be offloaded from resource-constrained sensors and bedside devices (including tens or hundreds of patient monitors and remote medical imaging (RMI) systems) to highly capable edge nodes that maintain rich context through DT-based representations of physical sensors and services. As many healthcare tasks are both computation-intensive and delay-sensitive [11], task offloading is essential to meet real-time requirements, while avoiding overload on local hospital devices. However, efficiently deciding where and when to offload tasks across heterogeneous hospital and edge servers remains challenging.

In reference [12], the task offloading problem becomes even more complex due to workload diversity, dynamic resource availability, queue fluctuations, and conflicting optimization goals such as latency reduction, energy efficiency and fair resource utilization. In reference [13], conventional scheduling methods, including static allocation strategies and simple heuristics like round-robin, are computationally lightweight but often struggle to adapt to the highly dynamic nature of healthcare environments. These approaches typically do not account for real-time system conditions such as CPU utilization, queue length, or communication quality. As a result, they may lead to inefficient resource use, increased delays, and even task drops. Similarly, energy-focused strategies can reduce power consumption but often at the cost of higher latency or uneven workload distribution across servers [14].

These limitations highlight the need for more intelligent scheduling mechanisms that can jointly optimize latency, energy consumption, fairness, and task reliability while still operating within practical system constraints. Another key challenge in DT-assisted edge systems lies in the accuracy and update frequency of digital twin synchronization. Since DT-based schedulers depend on estimated or predicted system states, outdated or fresh information can lead to suboptimal scheduling decisions, higher latency and inefficient resource utilization. On the other hand, overly frequent updates to DTs may introduce additional computational and communication overhead, especially when handling large volumes of data from multiple patient beds and remote imaging systems.

Existing work on DT-based edge computing has primarily focused on improving task performance metrics such as latency, throughput and reliability, often under the assumption of an ideal or cost-free digital twin layer [15]. In practice, however, building, synchronizing, and maintaining digital twins in dense sensing environments introduces non-negligible overheads. These include additional network traffic, processing costs, and control signaling at both core and edge nodes. When DT-driven workload placement decisions ignore these costs, the resulting solutions may be difficult to implement in real systems or may even offset the benefits that digital twins are expected to provide.

To address these limitations, this paper integrates a DT-informed mixed-integer linear programming (MILP) framework for task scheduling in healthcare edge computing environments. The framework jointly optimises task placement and resource allocation across heterogeneous hospital and edge servers while considering latency, energy consumption, and fairness. A network-edge digital twin is incorporated to capture short-term system states, including queue dynamics, resource utilization, and energy conditions, enabling informed offloading decisions. Unlike conventional predictors, such as time-series models (e.g., ARIMA) or machine-learning-based forecasters (e.g., LSTM networks), which estimate future system variables in an open-loop manner, the digital twin provides a continuously updated virtual representation of the healthcare edge system. The DT maintains a continuously updated representation of the system and functions as a control abstraction within the optimization loop. This allows explicit modeling of synchronization overhead and systematic evaluation of DT-related trade-offs under varying update policies. Furthermore, the framework examines the impact of DT state accuracy and update frequency on scheduling performance. Since offloading decisions rely on DT-maintained system states, discrepancies between physical and virtual systems may lead to inefficient or infeasible allocations. In delay-sensitive healthcare scenarios, such mismatches can increase waiting time, overload certain servers, or reduce fairness. Therefore, DT freshness is treated as a critical factor influencing system robustness, where both stale updates and excessive synchronization can negatively impact performance.

Simulation results show that the MILP-based scheduler significantly reduces average latency, eliminates task drops in the evaluated scenarios and improves load balancing across computing resources. Although total energy consumption increases moderately due to the higher task completion rate, the framework achieves better energy efficiency per completed task. In addition, the paper investigates the impact of digital twin staleness and update frequency. The results indicate that outdated DT states can degrade scheduling performance, whereas overly frequent synchronization introduces additional coordination overhead. Overall, these findings highlight the importance of jointly designing DT synchronization mechanisms and optimization-based scheduling strategies to achieve reliable and cost-efficient healthcare edge systems.

The main contributions of this paper are summarized as follows:

- We formulate DT edge *integration cost*, capturing coupled latency–energy–fairness effects when DT-driven workloads are offloaded across heterogeneous hospital and edge clinical servers.
- We propose a DT-informed MILP scheduling formulation that uses predicted edge states to make discrete offload and placement decisions under explicit system constraints, enabling traceable trade-off analysis rather than purely heuristic rules.
- We investigate the impact of model freshness (twinning rate) degradation and update frequency on system performance, showing that misaligned DT refresh cycles can adversely affect latency, admission control and fairness, while also increasing coordination overhead. These results motivate the co-design of twin update policies and optimization-based control mechanisms.

## 2. Related Work

### 2.1. Edge-Based Task Offloading

Energy efficiency is a major concern in edge-based task offloading, especially for medical wearables and IoT sensors with limited battery capacity [16]. Studies, such as [17], have focused on balancing uneven computational workloads in networks. These studies proposed task offloading strategies to reduce energy consumption and computation delay while maintaining high quality of service for users. For instance, ref. [18] presented optimal offloading schemes that significantly lower power consumption in battery-constrained environments. Likewise, ref. [19] combined heuristic techniques with machine learning to distribute computation across edge and cloud resources, leading to considerable energy savings. In instance [20], an energy-efficient adaptive scheduling framework was proposed, which jointly manages server resources and energy consumption. This improves resource utilization and QoS in heterogeneous fog computing environments.

Fairness in resource allocation is highlighted in distributed systems where multiple users or devices compete for limited computational and communication resources [21]. Load balancing addresses this by distributing tasks across servers to reduce response time and improve utilization. It is formulated as a bi-objective game balancing response time and fairness, and solved using a distributed load balancing algorithm (DLBA). Fairness-aware methods (e.g., reinforcement learning-based) have been proposed to dynamically adapt resource allocation under energy constraints and varying task priorities [22]. While promising, these approaches typically lack deterministic guarantees and often fail to explicitly capture the clinical urgency of healthcare workloads. Unlike generic edge offloading studies, healthcare-oriented task orchestration must account for heterogeneous clinical workloads, unequal urgency levels, etc. For instance, ECG monitoring, medical video analysis, and imaging tasks differ substantially in payload size, computation demand, and timeliness requirements, while some monitoring streams may be associated with alerts that are more critical than routine observations. Accordingly, the DT-assisted formulation in this paper is motivated by a healthcare setting in which reliability, deadline satisfaction, energy consumption, and balanced service continuity are analyzed.

There is plenty of previous work that targets the edge-based task offloading. Heuristic methods (FCFS, GA-only, LAHC) rely on simplified search rules or population-based exploration without explicit system modeling [23]. However, their reliance on local or stochastic search mechanisms inherently limits their ability to capture global system structure. Deep Reinforcement Learning (D3QN, DDQN, policy gradient methods) shifts toward direct policy learning, enabling adaptive decision-making in stochastic environments. However, despite their flexibility, DRL approaches suffer from two critical shortcomings: (i) the absence of formal optimality or safety guarantees, and (ii) poor robustness under distribution shifts or non-stationary environments. Additionally, their high sample complexity and training instability make them impractical for systems requiring rapid convergence or real-time guarantees [24]. MINLP solvers (BARON, ANTIGONE, Couenne) offer a more expressive formulation that can capture nonlinearities and integer constraints simultaneously. Despite this modeling richness, their exponential computational complexity severely restricts scalability. MILP represents a compromise between expressiveness and tractability, enabling globally optimal solutions under linearized assumptions with binary variables [24]. Overall, MILP offers an effective balance between optimality, interpretability, and modeling flexibility [23,25]. Although RL is more appropriate for highly dynamic environments and MINLP provides greater modeling accuracy for nonlinear systems, MILP remains the preferred optimization technique for many resource allocation applications due to its deterministic behavior, mature solver support, and guaranteed optimal solutions.

Table 1 highlights a comparison between the abovementioned approaches.

### 2.2. DT-Assisted Task Scheduling

Recent research has increasingly focused on DT-enabled optimization frameworks that jointly consider multiple system objectives [26]. In particular, several studies have demonstrated that integrating DTs with resource management can improve latency, energy efficiency and adaptability under dynamic operating conditions. For instance, ref. [27] proposed a multi-objective optimization model to balance energy consumption and latency, showing that joint optimization outperforms single-objective approaches. Similarly, ref. [28] introduced a context-aware framework that dynamically adapts resource allocation based on workload variations and environmental conditions, leading to improvements in both delay performance and energy efficiency.

Resource allocation and task offloading in DT-assisted systems have received significant attention in IoT and cyber-physical environments [29]. Existing state-of-the-art has primarily focused on individual objectives such as latency reduction, energy efficiency, or fairness under specific system constraints in [30]. While these works provide valuable insights, they often overlook the joint optimization of multiple conflicting performance metrics within a unified framework.

In the context of IoT-enabled healthcare networks, DTs are increasingly utilized as a network-level abstraction to support system monitoring and control rather than as direct enablers of clinical applications. The DT of the network deployed in [31] facilitates more informed task scheduling and resource allocation decisions by maintaining a virtual representation of the sensing and communication infrastructure. However, existing approaches typically do not fully capture the coupled trade-offs among latency, energy consumption, fairness and task reliability in such DT-assisted scheduling frameworks. In addition, the impact of DT-specific factors, such as synchronization overhead and freshness (twinning rate), is often neglected. To address these limitations, this work proposes a DT-informed MILP-based task scheduling framework that explicitly incorporates DT-derived system states while accounting for DT update dynamics, enabling efficient and reliable task offloading in IoT-based healthcare networks.

Conventional predictors and digital twins serve different roles in edge offloading systems. Traditional predictors, e.g., Auto-Regressive Integrated Moving Average (ARIMA), Support Vector Regression (SVR), Random Forest, Long Short-Term Memory (LSTM), and Gated Recurrent Unit (GRU), are widely used models for prediction and forecasting tasks [32]. Despite their effectiveness, these predictors operate in a feedforward manner, producing point estimates without maintaining an explicit representation of the physical system. As a result, their outputs are limited to prediction and must be externally integrated into decision-making processes. In contrast, the digital twin maintains a continuously synchronized virtual replica of the physical healthcare edge environment. Rather than forecasting isolated variables, DT integrates real-time data streams, historical information, and contextual system dynamics to track the system’s current state and simulate its evolution. This makes DT a system-level abstraction that supports monitoring, state consistency, and closed-loop scheduling decisions [33]. A further distinction lies in adaptability. Conventional predictors typically require retraining or recalibration when system conditions change, whereas DT can adapt through continuous synchronization with the physical infrastructure [6]. As a result, the DT is better suited for environments with rapidly varying workloads, heterogeneous edge resources, and stringent delay constraints, such as healthcare scenarios [28,34]. Overall, conventional predictors are appropriate for short-term forecasting, while DT is more suitable when prediction must be integrated with control, synchronization, and decision-making, as shown in Table 2.

## 3. Architecture of a Healthcare-Oriented Digital Twin System

**Figure 1:**
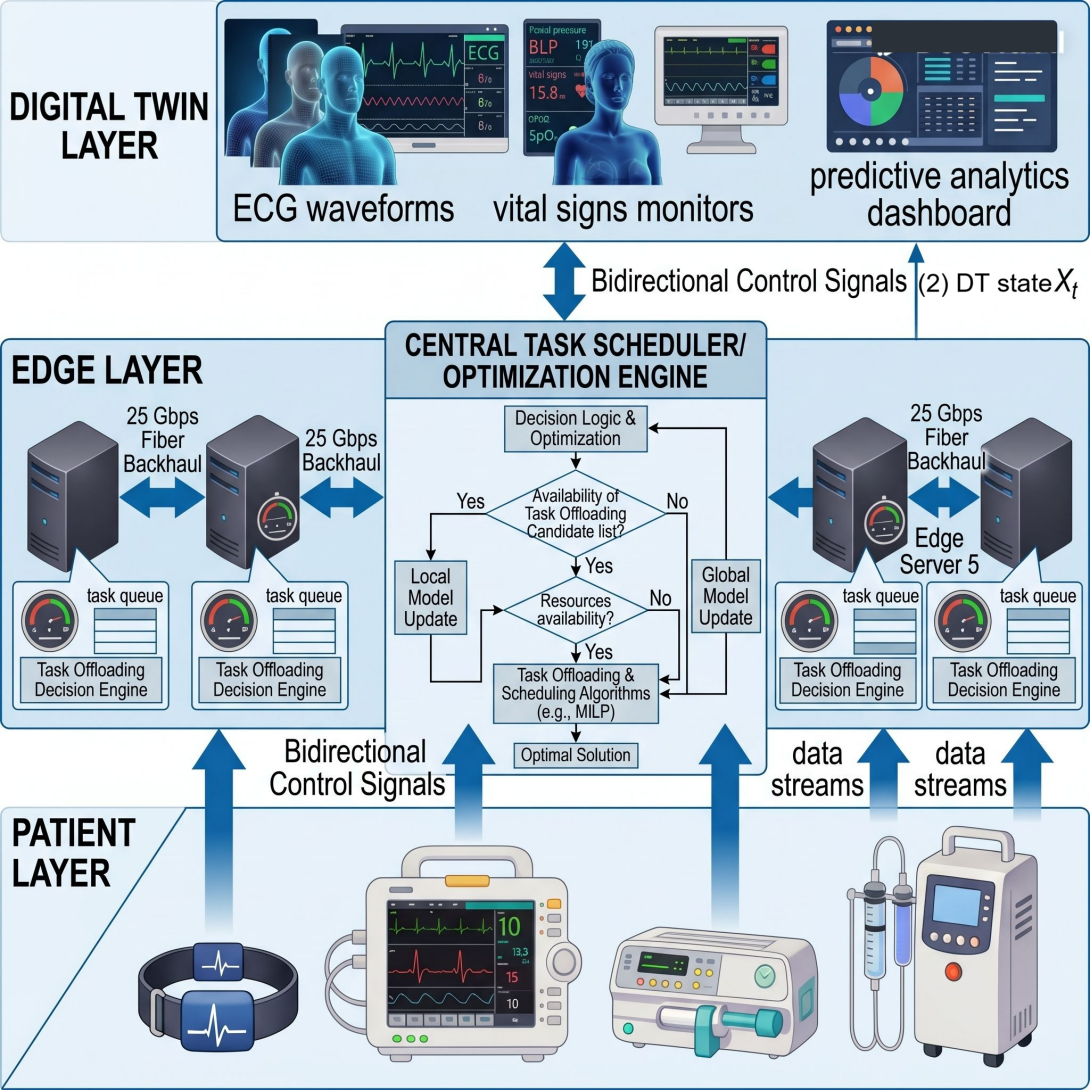
Illustrative architecture of a healthcare-oriented digital twin system.

Figure 1 illustrates the proposed healthcare-oriented digital twin architecture, which consists of three main layers: the patient layer, the edge layer and the DT Layer. These layers interact through bidirectional data and control flows to enable real-time monitoring, task offloading, and predictive healthcare analytics.

The patient layer includes wearable sensors and medical devices that continuously collect physiological data such as ECG signals, blood pressure, oxygen saturation and other vital signs. These data are transmitted to the nearest edge servers for processing (in the Edge Layer above).

The edge layer hosts multiple edge servers with task offloading and decision-making capabilities. These servers perform preliminary data processing, including buffering, filtering, denoising, and feature extraction. When a task is computationally intensive or delay-sensitive, the local offloading engine forwards it to a centralized task scheduler for global decision-making [35]. Edge servers communicate with each other through high-speed fiber backhaul links (as exemplified in [36]), enabling low-latency coordination and dynamic load balancing. The scheduler applies optimization techniques (e.g., MILP) to determine optimal task placement under resource, latency, and energy constraints.

The digital twin layer maintains virtual representations of patient physiological states and edge resource conditions using both real-time and historical data. This layer supports ECG visualization, vital sign tracking and predictive analytics. By continuously updating its models, the DT layer can generate proactive alerts [37] and assist clinicians in decision-making. The updated DT states are then fed back to the edge layer as control inputs, closing the loop between physical and virtual systems.

At the core of the architecture is a centralized task scheduler and optimization engine. This component coordinates decisions across the patient, edge, and DT layers by evaluating task requirements, checking resource availability, and updating both local and global system states. Its objective is to minimize latency and energy consumption while preserving fairness and maintaining consistency between predicted DT states and physical execution.

Overall, the proposed architecture models healthcare monitoring as a closed-loop control system. The patient layer generates continuous data streams, the edge layer handles latency-sensitive computation close to the data source, and the DT layer provides predictive intelligence to enhance scheduling and monitoring decisions [38]. This integration targets the enablement of a reliable, scalable and energy-aware healthcare service delivery.

## 4. System Model

The architectural elements described above are directly mapped to the system model to capture DT-assisted offloading decisions. In this work, the central hospital is modeled as the primary edge node within the edge layer, where tasks are initially generated and can also be executed locally. However, some healthcare tasks are delay-sensitive or computationally intensive and therefore cannot always be completed efficiently at the originating hospital node. To address this limitation, the edge layer also includes cooperating edge devices, such as nearby hospitals, clinics, or edge servers, which are interconnected through high-capacity fiber backhaul links and can receive migrated tasks from the central hospital. Accordingly, offloading refers to transferring a task from the central hospital to another cooperating edge node for execution, while local execution corresponds to processing the task at the originating hospital node. Based on this layered architecture, the proposed scheduling strategy is formulated as an MILP problem, which makes adaptive, system-aware decisions informed by both real-time edge conditions and DT predictions. In contrast, the hospital-only and round-robin schemes are used as baseline execution policies: the hospital-only scheme forces all tasks to remain at the central hospital, whereas the round-robin scheme assigns tasks cyclically across available cooperating edge nodes without considering resource availability, task urgency, or system state.

We consider a DT-enabled edge with *M* heterogeneous edge devices (each equipped with one CPU), interconnected via high-capacity fiber backhaul links within a multi-layer architecture: edge layer, DT layer, and task scheduler/optimization engine. Time is slotted with index t∈{1,…,T}. In each slot *t*, users deliver a total of $N_{t}$ tasks, where each task $i\in \{1,\ldots ,N_{t}\}$ appears initially on the edge device m∈{1,…,M} (e.g., the main hospital) and is characterized by the size of the input data $D_{i}^{t,m}$ (MB), the arrival time $T_{A,i}^{t,m}$, the estimated processing requirement $T_{P,i}^{t,m}$ (CPU cycles) and the strict deadline $T_{D,i}^{t,m}$ (s). Tasks can be (i) executed locally at edge device *m*, (ii) offloaded/migrated to another cooperating edge device n≠m over a constant-capacity backhaul, (iii) processed according to DT-predicted orchestration decisions, or (iv) dropped if infeasible under energy/latency constraints. We focus on real-time healthcare workloads and assume no long task queuing.

### 4.1. Task and Edge Device Model

For each task *i* at (t,m), we define binary decision variables

(1) $$x_{ij}^{t,m},\;\beta_{i}^{t,m},\;\gamma_{i}^{t,m},\;\delta_{i}^{t,m}\in \left\{0,1\right\},\;\beta_{i}^{t,m}+\gamma_{i}^{t,m}+\delta_{i}^{t,m}=1,\;\forall i,t,m,$$

where $\beta_{i}^{t,m}=1$ if task *i* is executed locally at device *m*, $\gamma_{i}^{t,m}=1$ if it is migrated to a remote edge device, and $\delta_{i}^{t,m}=1$ if it is dropped. When $\gamma_{i}^{t,m}=1$, the variable $x_{ij}^{t,m}=1$ selects a specific CPU $j\in \{1,\ldots ,J_{n}\}$ on destination edge device $n_{t}^{m}\neq m$; otherwise $x_{ij}^{t,m}=0$. The DT layer maintains a global state $X_{t}$ used by the scheduler to make predictive, system-aware decisions rather than purely reactive ones.

#### Destination CPU Selection of the Edge Device

If a task is migrated ($\gamma_{i}^{t,m}=1$), it must be assigned to exactly one CPU *j* on one destination edge device n≠m:

(2) $$\sum_{n\neq m}\sum_{j=1}^{J_{n}}x_{ij}^{t,m}=\gamma_{i}^{t,m},\;\forall i,t,m.$$

Each destination edge device *n* has CPU frequency $f_{\mathrm{edge}}^{n,j}$ (cycles/s) and per-MB cycle requirement $c_{\mathrm{edge}}^{n,j}$ for CPU *j*.

Offloading Decision: The offloading mode is selected by $\gamma_{i}^{t,m}$. When $\gamma_{i}^{t,m}=1$, task *i*’s data is transmitted over the fiber backhaul to edge device $n_{t}^{m}$ and processed on CPU *j*, instead of local execution ($\beta_{i}^{t,m}=1$) or dropping ($\delta_{i}^{t,m}=1$). This decision is optimized for every slot using DT state $X_{t}$, task features $\left\{D_{i}^{t,m},T_{P,i}^{t,m}\right\}$, and energy/latency constraints.

### 4.2. Latency Model

The end-to-end latency for all tasks is decomposed as

(3) $$L_{\mathrm{total}}=L_{\mathrm{comm}}+L_{\mathrm{exec}}+L_{\mathrm{comp}},$$

where $L_{\mathrm{comm}}$ is the communication latency over the fiber backhaul and DT signaling, $L_{\mathrm{exec}}$ is the execution time on edge CPUs, and $L_{\mathrm{comp}}$ captures the DT-driven scheduling computation overhead in the controller.

For a local task *i* at device *m*, the execution latency is

(4) $$T_{i,\mathrm{loc}}^{t,m}=\frac{T_{P,i}^{t,m}}{f_{t}^{m}}+T_{W,i}^{t,m},$$

where $f_{t}^{m}$ is the local CPU frequency (cycles/s) and $T_{W,i}^{t,m}$ is the waiting time on the selected CPU due to previously scheduled tasks.

For a migrated task, we assume a deterministic, constant-capacity fiber backhaul with rate $C_{\mathrm{backhaul}}$ (MB/s). The migration latency, including transmission and remote execution, is

(5) $$T_{i,\mathrm{mig}}^{t,m}=\frac{D_{i}^{t,m}}{C_{\mathrm{backhaul}}}+\frac{D_{i}^{t,m}c_{\mathrm{edge}}^{n,j}}{f_{\mathrm{edge}}^{n,j}}+T_{W,i}^{t,n},$$

where $T_{W,i}^{t,n}$ is the queueing delay at the destination edge CPU.

The actual task latency is then

(6) $$T_{i}^{t,m}=\beta_{i}^{t,m}T_{i,\mathrm{loc}}^{t,m}+\gamma_{i}^{t,m}T_{i,\mathrm{mig}}^{t,m},$$

subject to the deadline constraint

(7) $$T_{i}^{t,m}\leq T_{D,i}^{t,m}+M_{\mathrm{big}}\delta_{i}^{t,m},\forall i,t,m,$$

(8) $${\tilde{T}}_{i}^{t,m}=\min\left\{T_{i,\mathrm{loc}}^{t,m},\min_{n\neq m,j}T_{i,\mathrm{mig}}^{t,m}\left(n,j\right)\right\},$$

(9) $$\delta_{i}^{t,m}\leq 1\left\{{\tilde{T}}_{i}^{t,m}>T_{D,i}^{t,m}\right\},\forall i,t,m.$$

The CPU utilization of edge device *m* at time *t* is

(10) $$U_{\mathrm{CPU},m}^{t}=\frac{\sum_{i}L_{\mathrm{exec},i}^{t,m}}{T_{\mathrm{total},m}^{t}}\times 100\%,$$

where $L_{\mathrm{exec},i}^{t,m}$ is the execution time consumed by tasks on edge device *m* and $T_{\mathrm{total},m}^{t}$ is the total available processing time in slot *t*.

### 4.3. Energy Model

The total energy consumption of an edge device during task execution and offloading consists of two main components: (i) fiber transmission and (ii) computation at the executing server (local or remote). Fiber optic links are highly energy-efficient compared to copper-based interconnects, as their energy cost is dominated by the optical transceivers rather than cable length [38].

- Transmission Energy

The energy consumption for transmitting task *i* over a fiber backhaul link is mainly determined by the transceiver power of the optical interface modules:

(11) $$E_{\mathrm{tx},i}^{t,m}=P_{\mathrm{tx}}\cdot \frac{D_{i}^{t,m}}{C_{\mathrm{backhaul}}},$$

where $P_{\mathrm{tx}}$ (Watts) is the power of the optical transceiver, $D_{i}^{t,m}$ is the task data size (bits), and $C_{\mathrm{backhaul}}$ (bits/s) is the fiber link capacity. In typical deployments, $P_{\mathrm{tx}}\approx 1$ W for 10–25 Gbps optical modules, which makes fiber transmission highly energy-efficient [27].

- Computation Energy

The computation energy for executing task *i* at an edge device (local or remote) is modeled as the product of power and execution time:

(12) $$E_{\mathrm{comp},i}^{t,m}=P_{\mathrm{loc}}^{m}\cdot T_{i,\mathrm{loc}}^{t,m},$$

for local execution at device *m*, where $P_{\mathrm{loc}}^{m}$ is the average active power of device *m* and $T_{i,\mathrm{loc}}^{t,m}$ is given in Equation (4). Similarly, for remote execution at edge device *n* we have

(13) $$E_{\mathrm{comp},i}^{t,n}=P_{\mathrm{edge}}^{n}\cdot \frac{D_{i}^{t,m}c_{\mathrm{edge}}^{n,j}}{f_{\mathrm{edge}}^{n,j}},$$

where $P_{\mathrm{edge}}^{n}$ is the average active power of edge device *n*, $c_{\mathrm{edge}}^{n,j}$ is the per-MB cycle requirement, and $f_{\mathrm{edge}}^{n,j}$ the CPU frequency of CPU *j* on edge device *n*.

- Total Task Energy

The local execution energy of a task is given by

(14) $$E_{i,\mathrm{loc}}^{t,m}=E_{\mathrm{comp},i}^{t,m},$$

while the migration energy (transmission plus remote computation) is expressed as

(15) $$E_{i,\mathrm{mig}}^{t,m}=E_{\mathrm{tx},i}^{t,m}+E_{\mathrm{comp},i}^{t,n}.$$

The total energy consumed by edge device *m* in slot *t* is then

(16) $$E_{t}^{m}=\sum_{i}\left(\beta_{i}^{t,m}E_{i,\mathrm{loc}}^{t,m}+\gamma_{i}^{t,m}E_{i,\mathrm{mig}}^{t,m}\right),$$

where $\beta_{i}^{t,m}$ and $\gamma_{i}^{t,m}$ are binary indicators denoting local execution and migration decisions, respectively.

The evolution of the residual edge energy is defined as

(17) $$E_{t+1,m}=E_{t,m}-E_{t}^{m},\;E_{1,m}=E_{\mathrm{init},m},$$

subject to the reserve constraint

(18) $$E_{t,m}\geq \rho E_{\mathrm{init},m},\;0<\rho <1,\;\forall t,m.$$

### 4.4. Digital Twin Model

For each edge device *m*, the DT maintains a state vector $x_{t,m}$ that aggregates recent information about workload, CPU utilization, and residual energy. When a task is executed (locally or remotely), it contributes features $y_{t,m}$ extracted from its attributes (e.g., size, processing requirement, latency outcome):

(19) $$y_{t,m}=\phi \left(\left(\beta_{i}^{t,m}+\gamma_{i}^{t,m}\right)D_{i}^{t,m},T_{P,i}^{t,m},T_{i}^{t,m}\right),$$

where ϕ(·) is a feature-extraction operator.

The DT state is updated using an exponential moving average:

(20) $$x_{t+1,m}=\left(1-\eta \right)\;x_{t,m}+\eta \;y_{t,m}+\xi_{t,m},$$

where 0<η≤1 controls DT memory and $\xi_{t,m}$ captures external updates. The global DT state at time *t* is

(21) $$X_{t}=\left[x_{t,1},\ldots ,x_{t,M}\right],$$

and higher-level analytics are obtained via

(22) $$z_{t}=\Psi \left(X_{t}\right),$$

where Ψ(·) maps DT states to system indicators, such as predicted CPU utilisation and expected energy depletion, which the scheduler then uses for proactive offloading decisions.

To capture the effect of synchronization delay between the physical edge system and its virtual representation, we define the freshness of the digital twin state at time slot *t* as

(23) $$F_{t}=t-\tau_{t},$$

where $\tau_{t}$ denotes the most recent slot at which the DT state was successfully synchronized with the physical system. The corresponding DT staleness is defined as

(24) $$S_{t}=1-\exp\left(-\kappa F_{t}\right),$$

where κ>0 controls how rapidly the effect of outdated information grows. A larger $S_{t}$ indicates that the DT state is less representative of the current physical system. The freshness level directly affects scheduling quality. When the DT is fresh, the scheduler has access to more accurate queue, energy, and utilization estimates, which improves offloading decisions. When the DT becomes stale, prediction error increases, and the resulting MILP decisions may become suboptimal, leading to higher latency, unfair load distribution, or unnecessary task drops. To incorporate this effect, the DT analytics used by the scheduler are weighted by freshness as

(25) $$z_{t}=F_{t}\;\Psi \left(X_{t}\right),$$

or, equivalently, freshness may be introduced as a penalty term in the objective:

(26) $$J_{\mathrm{total}}=J+\lambda_{5}\sum_{t=1}^{T}S_{t}.$$

This formulation captures the trade-off between information accuracy and synchronization overhead. Very frequent updates reduce staleness but increase communication and computation cost, while infrequent updates reduce overhead but degrade decision quality.

### 4.5. Performance Metrics

Given the decision variables $\left\{\beta_{i}^{t,m},\gamma_{i}^{t,m},\delta_{i}^{t,m}\right\}$, we define the following:

- Total energy consumption:
  $$E_{\mathrm{used}}=\sum_{t=1}^{T}\sum_{m=1}^{M}E_{t}^{m}.$$
- Task drop rate:
  $$\mathrm{DropRate}=\frac{\sum_{t=1}^{T}\sum_{m=1}^{M}\sum_{i}\delta_{i}^{t,m}}{\sum_{t=1}^{T}\sum_{m=1}^{M}N_{t}}.$$
- Average latency (over successfully served tasks):
  $$\bar{T}=\frac{\sum_{t=1}^{T}\sum_{m=1}^{M}\sum_{i}T_{i}^{t,m}\left(1-\delta_{i}^{t,m}\right)}{\sum_{t=1}^{T}\sum_{m=1}^{M}\sum_{i}\left(1-\delta_{i}^{t,m}\right)}.$$
- Average CPU utilization:
  $${\bar{U}}_{\mathrm{CPU}}=\frac{1}{MT}\sum_{t=1}^{T}\sum_{m=1}^{M}U_{\mathrm{CPU},m}^{t}.$$
- Energy fairness across devices using Jain’s index, where $E_{m}=\sum_{t=1}^{T}E_{t}^{m}$:
  $$\mathrm{FI}=\frac{{\left(\sum_{m=1}^{M}E_{m}\right)}^{2}}{M\sum_{m=1}^{M}E_{m}^{2}}.$$

### 4.6. Optimization Problem

The DT-driven controller jointly optimizes task execution decisions $\left\{\beta_{i}^{t,m},\gamma_{i}^{t,m},\delta_{i}^{t,m}\right\}$, CPU assignment $x_{ij}^{t,m}$, and backhaul usage to balance energy, latency, reliability, and fairness. The multi-objective cost over horizon *T* is

(27) $$\begin{array}{cc} \min_{\left\{x,\beta ,\gamma ,\delta \right\}}\;J= & \;\lambda_{1}\sum_{t=1}^{T}\sum_{m=1}^{M}\sum_{i}∥T_{i}^{t,m}∥+\lambda_{2}\sum_{t=1}^{T}\sum_{m=1}^{M}∥E_{t}^{m}∥\\ \; & +\lambda_{3}\sum_{t=1}^{T}\sum_{m=1}^{M}\sum_{i}∥\delta_{i}^{t,m}∥+\lambda_{4}{∥1-\mathrm{FI}∥}_{\max}, \end{array}$$

where $\lambda_{1}+\lambda_{2}+\lambda_{3}+\lambda_{4}=1$ and ∥·∥ denotes absolute value. The problem is subject to Constraints (1)–(9), energy reserve constraints, and binary variable domains. This MINLP can be solved offline via MILP solvers (intlinprog) or approximated online via DRL, where the action space is $\left\{\beta_{i}^{t,m},\gamma_{i}^{t,m},\delta_{i}^{t,m}\right\}$ conditioned on the DT state $X_{t}$, enabling energy-aware and deadline-compliant load balancing over the fiber-connected edge devices. Subject to

(28) $$\beta_{i}^{t,m}+\gamma_{i}^{t,m}+\delta_{i}^{t,m}=1,\forall i,t,m,$$

(29) $$\sum_{n\neq m}\sum_{j=1}^{J_{n}}x_{ij}^{t,m}=\gamma_{i}^{t,m},\forall i,t,m,$$

(30) $$T_{i}^{t,m}\leq T_{D,i}^{t,m}+M_{\mathrm{big}}\delta_{i}^{t,m},\forall i,t,m,$$

(31) $$\delta_{i}^{t,m}\leq 1\left\{{\tilde{T}}_{i}^{t,m}>T_{D,i}^{t,m}\right\},\forall i,t,m,$$

(32) $$\sum_{i}x_{ij}^{t,m}\frac{D_{i}^{t,m}c_{\mathrm{edge}}^{m,j}}{f_{\mathrm{edge}}^{m,j}}\leq \Delta t,\forall m,j,t,$$

(33) $$\sum_{m=1}^{M}\sum_{i:\gamma_{i}^{t,m}=1}\frac{D_{i}^{t,m}}{\Delta t}\leq C_{\mathrm{backhaul}},\forall t,$$

(34) $$E_{t+1,m}=E_{t,m}-E_{t}^{m},\;E_{1,m}=E_{\mathrm{init},m},\forall m,$$

(35) $$E_{t,m}\geq \rho E_{\mathrm{init},m},\forall t,m,$$

(36) $$\beta_{i}^{t,m},\gamma_{i}^{t,m},\delta_{i}^{t,m}\in \left\{0,1\right\},\forall i,m,t.$$

Problem (27)–(36) defines a digital-twin-driven edge computing optimization framework for healthcare applications.

The problem is NP-hard due to binary variables and nonlinear energy/latency terms but admits efficient offline solutions via MILP solvers (Gurobi/intlinprog) over short horizons or online approximations via model-free DRL (DDPG) conditioned on DT analytics $z_{t}=\Psi \left(X_{t}\right)$, enabling real-time, energy/latency/fairness-aware task orchestration across heterogeneous edge servers while ensuring clinical deadlines are met or gracefully violated only when physically infeasible.

This MILP formulation advances the state-of-the-art by (i) integrating DT state prediction $X_{t}$ into the optimization loop for proactive load balancing rather than reactive scheduling, (ii) jointly modeling CPU assignment $x_{ij}^{t,m}$, execution modes $\left\{\beta_{i}^{t,m},\gamma_{i}^{t,m},\delta_{i}^{t,m}\right\}$ and resource constraints under a unified multi-objective framework, and (iii) providing deadline-aware task dropping that respects clinical urgency while maintaining energy sustainability (35) and backhaul capacity (33).

## 5. Algorithm for Joint Task Offloading and Resource Allocation

Section 5 provides a high-level description of the proposed DT-assisted MILP scheduling and optimization algorithms to illustrate the overall workflow of the framework. The detailed mathematical formulation, system assumptions, variables, constraints, and parameter definitions used by these algorithms are presented in Section 4. The algorithmic procedures introduced in this section should therefore be interpreted as conceptual representations, with their formal definitions and analytical foundations provided in the following section.

To solve the formulated problem, we propose an iterative DT-assisted decision-making algorithm, detailed in Algorithms 1 and 2, which determines the execution mode (local computation, migration, or task drop) across edge servers at each time slot.
**Algorithm 1** Overall DT-Assisted Edge Computing System with Freshness Awareness**Require:** 
$M,\;T,\;\Delta t,\;C_{\mathrm{backhaul}},\;\left\{f_{t}^{m},\;f_{\mathrm{edge}}^{m,j},\;c_{\mathrm{edge}}^{m,j}\right\}$, $E_{\mathrm{init},m},\;\eta ,\;\rho ,\;\kappa ,\;\epsilon_{\mathrm{stale}}$**Ensure:** 
Decisions $\left\{\left(\beta_{i}^{t,m},\gamma_{i}^{t,m},\delta_{i}^{t,m}\right)\right\}$  1:Initialize $E_{1,m}\leftarrow E_{\mathrm{init},m},\forall m$  2:Initialize $x_{1,m}\leftarrow 0,\forall m$, $\tau_{1}\leftarrow 1$  3:$X_{1}\leftarrow \left[x_{1,1},\ldots ,x_{1,M}\right]$  4:**for** t=1 to *T* **do**  5:      Observe incoming tasks $\left\{D_{i}^{t,m},T_{P,i}^{t,m},T_{D,i}^{t,m}\right\}$  6:      Compute freshness: $F_{t}\leftarrow t-\tau_{t}$  7:      Compute staleness: $S_{t}\leftarrow 1-\exp\left(-\kappa F_{t}\right)$  8:      **if** $S_{t}>\epsilon_{\mathrm{stale}}$ **then**  9:           Synchronize DT with physical system10:           $\tau_{t}\leftarrow t$11:           Update $X_{t}$12:      **end if**13:      $z_{t}\leftarrow F_{t}\Psi \left(X_{t}\right)$                ▷ Freshness-weighted DT analytics14:      **for** each task *i* at server *m* **do**15:           Compute local latency $T_{i,\mathrm{loc}}^{t,m}$ using (4)16:           Compute migration latency $T_{i,\mathrm{mig}}^{t,m}\left(n,j\right)$ using (5)17:           ${\tilde{T}}_{i}^{t,m}\leftarrow \min\left\{T_{i,\mathrm{loc}}^{t,m},\min_{n\neq m,j}T_{i,\mathrm{mig}}^{t,m}\left(n,j\right)\right\}$18:           **if** ${\tilde{T}}_{i}^{t,m}>T_{D,i}^{t,m}$ **then**19:                $\delta_{i}^{t,m}\leftarrow 1$, $\beta_{i}^{t,m}\leftarrow 0$, $\gamma_{i}^{t,m}\leftarrow 0$20:                **continue**21:           **end if**22:           **if** ${\mathrm{Trigger}}_{\mathrm{offload}}^{t,m}=1$ **then**23:                ProposedMILP($z_{t},S_{t},i,m$)24:           **else**25:                $\beta_{i}^{t,m}\leftarrow 1$ if $U_{\mathrm{CPU},m}^{t}<{\bar{U}}_{\mathrm{CPU}}^{t}-\sigma$26:           **end if**27:      **end for**28:      Compute total latency $T_{i}^{t,m}$ using (6)29:      Update energy:$$E_{t}^{m}\leftarrow \sum_{i}\left(\beta_{i}^{t,m}E_{i,\mathrm{loc}}^{t,m}+\gamma_{i}^{t,m}E_{i,\mathrm{mig}}^{t,m}\right)$$30:      $E_{t+1,m}\leftarrow \max\left(E_{t,m}-E_{t}^{m},\rho E_{\mathrm{init},m}\right),\forall m$31:      Update fairness:$${\bar{U}}_{\mathrm{CPU}}^{t}\leftarrow \frac{1}{M}\sum_{m}U_{\mathrm{CPU},m}^{t}$$32:      **for** each server *m* **do**33:           Extract features:$$y_{t,m}\leftarrow \phi \left(\left(\beta +\gamma \right)D,T_{P},T\right)$$34:           Update DT state:$$x_{t+1,m}\leftarrow \left(1-\eta \right)x_{t,m}+\eta y_{t,m}+\xi_{t,m}$$35:      **end for**36:      $X_{t+1}\leftarrow \left[x_{t+1,1},\ldots ,x_{t+1,M}\right]$37:**end for**38:**return** all scheduling decisions

**Algorithm 2** DT-MILP Task Offloading
**Require:** 
$z_{t},S_{t}$, task *i* at *m*, $\left\{f_{\mathrm{edge}}^{n,j},c_{\mathrm{edge}}^{n,j}\right\}$, λ,ρ**Ensure:** 


$\left(\beta_{i}^{t,m},\gamma_{i}^{t,m},\delta_{i}^{t,m}\right)$


  1:Formulate MILP objective:$$J=\lambda_{1}T_{i}^{t,m}+\lambda_{2}E_{t}^{m}+\lambda_{3}\delta_{i}^{t,m}+\lambda_{4}\left(1-\mathrm{FI}\right)+\lambda_{5}S_{t}$$  2:Subject to constraints:(28)–(36)  3:Warm-start using DT predictions:$$z_{t}\to \left\{U_{\mathrm{CPU}},E_{t},\mathrm{fairness}\;\mathrm{indicators}\right\}$$  4:Solve MILP:$$\left(\beta_{i}^{t,m},\gamma_{i}^{t,m},\delta_{i}^{t,m}\right)\leftarrow \mathrm{intlinprog}\left(J\right)$$  5:**if** $\gamma_{i}^{t,m}=1$ **then**  6:      Select destination:$$\left(n^{🟉},j^{🟉}\right)\leftarrow \arg\max x_{ij}^{t,m}$$  7:      Offload task to CPU $j^{🟉}$ at server $n^{🟉}$  8:
**end if**
  9:**return** optimal decision variables


Algorithm 1 presents the overall workflow of the proposed DT-assisted edge computing framework with freshness awareness. At the beginning of each scheduling interval, the system initializes the DT states, energy levels, and decision variables for all edge servers. For every time slot, newly arriving tasks are observed, and the freshness and staleness of the DT are evaluated based on the elapsed synchronization time. When the freshness falls below a predefined threshold, the DT is synchronized with the physical system to update the system state before performing scheduling decisions. The refreshed DT is then used to extract system features and estimate task execution performance. For each task-server pair, the algorithm computes the local execution latency and migration latency, selects the minimum feasible execution time, and checks whether the task satisfies its delay deadline. If the deadline cannot be met, the task is dropped; otherwise, the algorithm determines whether offloading is required. Offloading decisions are optimized through the MILP formulation while considering server resource availability and CPU utilization fairness. After the scheduling decisions are obtained, the total task latency and energy consumption are calculated, and the remaining battery energy is updated subject to a minimum energy constraint. Finally, server features are extracted to update the DT states using the system dynamics model, and the refreshed DT is carried forward to the next scheduling interval.

Algorithm 2 describes the proposed DT-MILP task offloading strategy with freshness awareness. Given the current DT state, system status, task information, edge server resources, and optimization weights, the algorithm first formulates a multi-objective MILP that jointly minimizes task latency, energy consumption, task rejection, fairness degradation, and DT staleness through a weighted objective function. The optimization is performed subject to the system constraints defined in Equations (28)–(36). To improve computational efficiency and solution quality, the MILP is warm-started using DT-predicted system information, including CPU utilization, residual energy, and fairness indicators. The resulting optimization is to determine the optimal task admission, offloading, and execution decisions. If a task is selected for offloading, the algorithm identifies the optimal destination server and CPU core according to the optimization outcome and assigns the task accordingly. Finally, the optimal decision variables are returned to the DT-driven scheduling framework, enabling adaptive and freshness-aware task offloading while balancing latency, energy efficiency, resource utilization, and fairness across edge servers.

## 6. Results and Discussion

### 6.1. Simulation Setup

Simulation was conducted in MATLAB R2025a on a workstation equipped with an Intel Core i9 CPU and 64 GB RAM. The considered scenario is a DT-enabled heterogeneous edge network for healthcare monitoring, consisting of *M* = 5 edge servers, consisting one hospital and multiple cooperating clinics. Such a configuration is commonly acknowledged in plenty of European countries, where the (public) hospitals and clinics within the city form a local edge network. The public hospital suffers from overloading, whereas the clinics have spare compute resources that could be utilized to release the hospital’s workload [39]. Time is discretized into slots of Δt = 1 ms over *T* = 2000 slots, with one to five tasks arriving per slot and being drawn uniformly from ECG, video, and medical imaging with heterogeneous input sizes and clinical deadlines as mentioned in Table 3. This one-to-five-arrival-per-slot configuration is used as a controlled starting point for isolating the impact of DT-assisted scheduling under heterogeneous tasks. Task attributes are generated as follows: payload size $D_{i}^{t,1}∼U\left[1,5\right]$ MB, per-byte computational demand $c_{\mathrm{edge}}^{n,j}∼U\left[800,1500\right]\times 10^{6}$ cycles/byte, and clinical deadlines $T_{D,i}^{t,1}∼U\left[2,20\right]$ slots (20–200 ms). CPU frequencies are sampled as $f_{t}^{m}∼U\left[1.5,2.5\right]$ GHz for the hospital and $f_{t}^{m}∼U\left[2,3\right]$ GHz for clinic servers. Initial residual energies are set to $E_{\mathrm{init},m}\in \left[1000,2000\right]$ J with a reserved fraction of ρ=0.25. Processing power of each CPU is set in a range of 160–250 W. The servers are connected via a fiber backhaul link with a fixed capacity of $C_{\mathrm{backhaul}}=500$ MB/s, which is common in healthcare local networks [40,41]. The DT layer forecasts short-term CPU utilization and energy states using an exponential moving average with smoothing factor η=0.1. We compare the proposed DT-informed MILP scheduler against hospital-only and round-robin (RR) baselines, using a consistent weight vector λ=[0.25] across all methods. Performance is evaluated in terms of mean end-to-end latency $\bar{L}$, total energy consumption $E_{\mathrm{used}}$, task drop rate, Jain’s fairness index, and hospital overload reduction.

### 6.2. System-Level Performance

As summarized in Table 4, the hospital-only baseline reveals the limitations of relying exclusively on local edge resources for healthcare task execution. Since all incoming tasks are processed by the hospital server, the node rapidly becomes overloaded, leading to a task drop rate of 84.30% and a Jain’s Fairness Index (JFI) of only 0.200. These results indicate that a single edge server cannot accommodate the continuous and computationally intensive workload generated by modern healthcare monitoring applications, highlighting the need for cooperative resource sharing across multiple edge nodes.

The RR scheduler partially addresses this limitation by distributing tasks among the available edge servers. This strategy increases the number of successfully processed tasks to 4030 while reducing the task drop rate to 59.70%. However, the corresponding fairness index remains relatively low (0.436), suggesting that static cyclic scheduling is unable to adapt effectively to heterogeneous server capabilities and dynamically changing workloads. Because RR assigns tasks without considering current queue lengths, processing loads, or resource availability, it continues to experience resource imbalance and congestion under heavy traffic conditions.

In contrast, the proposed DT-enabled MILP scheduler consistently delivers the best performance across all evaluated metrics. By incorporating real-time system monitoring and predictive state information from the DT, the scheduler successfully processes all 10,000 tasks, achieving a task drop rate of 0.00%. In addition, it attains a fairness of 0.907, demonstrating a highly balanced distribution of computational workload among the participating edge servers. These results demonstrate that integrating DT technology with global optimization not only eliminates task loss but also significantly improves fairness in resource allocation. Such capabilities are particularly important in edge computing in healthcare, where timely task execution and uninterrupted service availability are essential for supporting mission-critical applications.

The latency results presented in Table 4 indicate that the average end-to-end delay per processed task remains nearly identical across all scheduling approaches, at approximately 10.34 ms. This suggests that once a task is admitted for execution, the communication and processing delays are comparable regardless of the scheduling strategy.

However, per-task latency alone does not fully capture overall system performance. A more meaningful distinction emerges when considering the total delay and the number of successfully completed tasks as shown in Table 5. The MILP scheduler accumulates a higher total delay (97,812.35 ms) than RR (42,577.35 ms) and hospital-only (16,240.77 ms) because it successfully processes a substantially larger number of tasks. In contrast, RR and hospital-only exhibit lower aggregate delay primarily because many tasks are dropped and therefore do not contribute to the delay statistics.

From a healthcare perspective, the MILP-based scheduler is the preferred solution because it maintains low per-task latency while achieving significantly higher task completion rates. These results demonstrate that the scheduler can sustain service quality without sacrificing responsiveness, ensuring that a greater proportion of healthcare requests are successfully served. Consequently, the MILP approach provides a more effective balance between latency performance, service availability, and workload handling capability, all of which are critical requirements in healthcare edge-computing systems.

The energy results highlight an important distinction between total energy consumption and energy efficiency. Although the MILP-based scheduler exhibits the highest total energy consumption due to processing the full set of 10,000 tasks, it is the most efficient in terms of normalized energy per task. Specifically, the average task energy is 0.615 J/task for MILP, compared with 0.907 J/task for round-robin (RR) and 1.599 J/task for hospital-only as shown in Table 6.

This indicates that lower total energy in the baselines does not reflect better efficiency, but rather results from processing fewer tasks and dropping a significant portion of the workload. In contrast, MILP achieves lower energy per task while completing the full workload, making it a more appropriate measure of energy efficiency in healthcare edge systems.

A similar trend is observed in power consumption. MILP maintains the lowest average power at 95.13 W, followed by RR at 110.60 W, while hospital-only reaches 154.53 W as shown in Table 4. This confirms that optimization-based task placement reduces power stress and prevents the inefficient operation associated with single-node congestion.

The results reveal a clear trade-off between fairness and energy consumption. The hospital-only strategy exhibits the lowest fairness (0.200) because all tasks are executed on a single server. RR improves fairness (0.436) through workload distribution, but its static scheduling policy cannot adapt to dynamic resource conditions. Although MILP without DT employs optimization, its fairness remains limited (0.300) due to the lack of real-time system awareness. In contrast, the proposed DT-enabled MILP framework achieves the highest fairness (0.907) by dynamically balancing workloads using digital twin-assisted resource information (Table 4).

While the DT-enabled MILP scheduler incurs the highest total energy consumption (Table 6), this is primarily because it successfully processes all 10,000 tasks, unlike the baseline approaches that drop a substantial portion of the workload. Therefore, the increase in total energy reflects higher system productivity rather than lower efficiency. In mission-critical healthcare applications, this trade-off is justified, as ensuring uninterrupted service, eliminating task loss, and maintaining balanced resource utilization are more important than minimizing aggregate energy consumption alone.

To strengthen the analysis beyond the baseline setting, the server-scaling results for 5 and 10 edge servers are used to evaluate how reliability, fairness, and energy efficiency change with infrastructure size. This additional analysis is important because the mathematical model permits multiple tasks per slot and heterogeneous server deployments, whereas a single fixed operating point would not fully demonstrate the robustness of the scheduling framework. Table 7 compares the scenarios with 5 and 10 edge servers, based on the same setup in Table 3. Both configurations process all the 10,000 tasks with zero drops, but the five-server setup achieves superior energy efficiency and fairness. It consumes 36% less total system energy (6149.93 J vs. 9564.01 J), attains a significantly higher Jain’s fairness index (0.907 vs. 0.376, because of more idle states of CPUs), and maintains lower average total power (73.01 W vs. 106.96 W). The 5-server configuration also demonstrates more effective CPU load management with an average offload CPU requirement of 72.85% compared to 49.99% for 10 servers. While the 10-server configuration shows marginally lower average end-to-end delay (10.3406 ms vs. 10.3417 ms), it incurs substantially higher energy costs and poorer load balancing across servers. These results demonstrate that increasing server count does not guarantee better performance. This finding is particularly relevant for healthcare edge computing scenarios, where energy-constrained and latency-sensitive applications require efficient task scheduling. The framework’s ability to maintain high task completion rates, low energy consumption, and balanced resource utilization demonstrates its potential for deployment in large-scale, dynamic healthcare environments, ultimately supporting a more sustainable and reliable edge-based healthcare infrastructure.

### 6.3. Digital Twin Update Frequency and Scalability Trade-Offs

This section evaluates the impact of DT update frequency on key system metrics, including end-to-end latency, DT waiting latency, task drop rate, energy consumption, and workload fairness.

To start with, an ablation study w/o DT is analyzed. Without real-time knowledge of resource availability and system dynamics, the MILP-only scheduler executed 4994 out of 10,000 tasks, resulting in a 50.01% task drop rate. In addition, it achieved a Jain’s fairness index of only 0.300, indicating an imbalanced distribution of workload across the available computing resources, and consumed an average of 0.894873 J per processed task. Although the average task delay remained relatively low at 10.314 ms for successfully executed tasks, the high task rejection rate and poor resource utilization demonstrate the limitations of static system information. Integrating a DT overcomes these shortcomings by maintaining an up-to-date virtual representation of the physical infrastructure, enabling more informed scheduling decisions. However, the effectiveness of the DT depends on its synchronization frequency relative to the physical system. While more frequent updates can improve scheduling accuracy and resource allocation, they also incur additional communication, computation, and synchronization overhead. Therefore, it is essential to investigate the trade-off between DT update frequency, system scalability, and overall scheduling performance.

**Figure 2:**
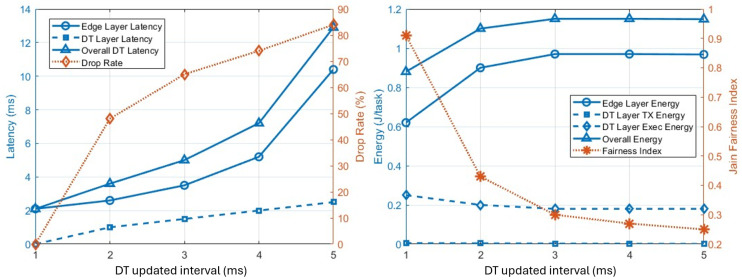
Digital twin system performance comparison for updated frequency 1, 2, 3, 4, and 5 ms.

Figure 2 illustrates the impact of the DT update interval on state estimation errors and cost of DT-assisted scheduling, where the MILP optimizer relies on DT-provided system states such as server utilization, queue length, and communication delays. The DT update interval is varied from 1 to 5 ms, while all other system parameters are kept fixed, in order to isolate the effect of more frequent DT synchronization on system performance. At a short update interval (1 ms), frequent synchronization enables the DT to maintain accurate and up-to-date system states, allowing the scheduler to achieve the lowest overall latency (2.1 ms), zero task drop rate, and the highest fairness (Jain’s index of 0.91) while maintaining relatively low energy consumption. As the update interval increases from 2 ms to 5 ms, synchronization becomes less frequent, resulting in progressively stale state information and larger state estimation errors. Consequently, the MILP scheduler makes increasingly suboptimal resource allocation decisions, leading to a steady increase in edge-layer, DT-layer, and overall latency, with the overall latency reaching 12.9 ms at a 5 ms update interval. Meanwhile, the task drop rate rises significantly to 84%, reflecting the growing number of tasks that miss their execution deadlines under outdated system information. In addition, the energy trend changes only moderately, with edge layer energy increasing from about 0.61 J/task to 0.96 J/task and overall energy reaching roughly 1.13 J/task at the highest update interval. DT execution energy remains close to 0.25 J/task at an interval of 1 ms and gradually decreases to about 0.18 J/task, while DT TX energy drops from approximately 0.10 J/task to about 0.00 J/task. In contrast, fairness, as Jain’s index declines from 0.907 to 0.255. It suggests that excessively frequent updates cause the scheduler to overburden a small number of servers while leaving others underutilized. Overall, these results demonstrate that frequent DT synchronization is essential for maintaining accurate system state awareness and achieving low latency, low task drop rate, high fairness, and improved energy efficiency, whereas excessively long update intervals significantly degrade scheduling performance.

## 7. Conclusions

The results demonstrate that static non-cooperative and cyclic scheduling policies are not well suited to heterogeneous healthcare edge environments with continuous task arrivals. In contrast, the proposed DT-assisted MILP scheduler eliminates task loss, achieves higher fairness, reduces per-task energy consumption, and lowers mean total power while maintaining clinically acceptable end-to-end latency.

The analysis of DT update frequency further reveals an important trade-off between responsiveness and stability. Increasing the update frequency reduces execution latency by providing more accurate and timely system information. However, it also increases synchronization overhead, task drop rates, and load imbalance across servers. These findings indicate that excessively frequent updates can degrade overall system reliability despite improving nominal latency.

For healthcare DT-enabled edge systems, moderate update frequencies combined with adaptive scheduling mechanisms provide a more effective balance between latency, reliability, and fairness. Overall, the results highlight that integrating digital twin intelligence with optimization-based scheduling is essential for achieving reliable, energy-efficient, fair, and scalable healthcare edge computing.

## Figures and Tables

**Table 1:** Structural and functional comparison of representative optimization frameworks.

Technique	Name(s)	Pros	Cons	Limitations
Heuristic	FCFS, GA-only, LAHC	Simple implementation.	Often converges to local optima and may fail to identify globally optimal solutions.	Limited awareness of detailed system states and poor scalability under heterogeneous workloads.
DRL-based	D3QN, DDQN, Policy Gradient	Learns adaptive scheduling policies suitable for dynamic environments.	Requires extensive training and may become unstable under changing data distributions.	Provides no deterministic optimality guarantees and lacks formal safety certification.
MINLP	BARON, ANTIGONE, Couenne	Highly expressive mathematical modeling capable of representing nonlinear interactions.	Computationally expensive and often exhibits slow convergence.	Generally unsuitable for real-time scheduling due to excessive solution time.
MILP	DT-MILP	Combines digital twin awareness with MILP optimality, enabling adaptive, fair, and globally optimal scheduling decisions.	Introduces additional computational overhead for digital twin synchronization and optimization.	Performance depends on accurate digital twin synchronization and timely state updates.

**Table 2:** Comparison between digital twin and conventional predictors.

Aspect	Time-Series/ML Predictors	Digital Twin (DT)
System Representation	Uses historical data to learn patterns; no explicit system replica	Maintains a continuously synchronized virtual replica of the physical system
Output Type	Point estimates (e.g., latency, workload, energy)	Real-time state estimation + future behavior simulation
Data Usage	Primarily past observations	Real-time data streams + historical + contextual system dynamics
Adaptability	Limited; requires retraining for changes	Adaptive through continuous synchronization
Decision Support	Supports prediction only (feedforward)	Enables closed-loop control and decision-making
Model Examples	ARIMA, SVR, Random Forest, LSTM, GRU	Physics-based models + data-driven hybrid system models
Temporal Awareness	Strong in short-term forecasting (especially ML models)	Strong in both short-term prediction and dynamic evolution tracking
Interaction with System	Passive predictor	Active system-aware emulator (bi-directional feedback)

**Table 3:** Representative parameter ranges for edge CPU allocation (simulation setup).

Parameter	Value(s)
Number of edge servers *M*	5/10
Number of tasks	$10^{4}$
Average task arrival time per slot	1–5
Average Updated Frequency	1–5 ms
*T*	2000 slots
Slot length Δt	1 ms
Backhaul capacity $C_{\mathrm{backhaul}}$	500 MB/s
Reserved energy ratio ρ	0.25
Mean CPU frequency $f_{m}$	2.0–3.0 GHz
Mean processing power $P_{m}$	160–250 W
Mean initial energy $E_{m}^{\mathrm{init}}$	1000–2000 J
Mean ECG/Video/Imaging sizes	90/280/150 KB

**Table 4:** Performance comparison of DT-enabled MILP, round-robin (RR), and hospital-only scheduling.

Method	Executed Tasks	Drop (%)	Fairness	Avg Delay (ms)	Avg Energy (J)
DT–MILP	10,000	0.00	0.907	10.3417	0.615000
RR	4030	59.70	0.436	10.3393	0.907000
Hospital-only	1570	84.30	0.200	10.3444	1.599000

**Table 5:** Aggregate delay across processed tasks for DT-enabled MILP, RR, and hospital-only scheduling.

Delay Metric (ms)	DT–MILP	RR	Hospital-Only
Total TX Delay	3232.3496	2277.3467	540.7748
Total Exec Delay	94,580.0000	40,300.0000	15,700.0000
Total Task Delay	97,812.3496	42,577.3467	16,240.7748

**Table 6:** Energy comparison across processed tasks for DT-enabled MILP, RR, and hospital-only scheduling.

Energy Metric (J)	DT–MILP	RR	Hospital-Only
Total TX Energy	17.082833	6.837233	2.703874
Total Exec Energy	6132.845000	3647.285000	2507.050000
Total Task Energy	6149.927833	3654.122233	2509.753874
Total System Energy	6149.927833	3654.122233	2509.753874
Average TX Energy per Task	0.001708	0.001697	0.001722
Average Exec Energy per Task	0.613284	0.905033	1.596847
Average Task Energy per Task	0.614993	0.906730	1.598569

**Table 7:** Comparison of overall system performance for different numbers of edge servers.

Metric	5 Servers	10 Servers
Total Tasks Generated	10,000	10,000
Successfully Assigned	10,000	10,000
Tasks Dropped	0	0
Drop Rate (%)	0.00	0.00
Total TX Delay (ms)	3416.57	3415.5230
Total Execution Delay (ms)	100,000	99,990
Total End-to-End Delay (ms)	103,405.5230	103,416.57
Average End-to-End Delay (ms)	10.3417	10.3406
Average Jain Fairness Index	0.907	0.376
Highest CPU Utilization (%)	100	60.0
Average Total Power (W)	73.01	106.96
Total TX Energy (J)	17.083	17.083
Total Execution Energy (J)	6132.845	9546.928
Total System Energy (J)	6149.928	9564.011
Average TX Energy per Task (J)	0.001708	0.001708
Average Execution Energy per Task (J)	0.613284	0.954693
Average Total Energy per Task (J)	0.614993	0.956401

## Data Availability

The raw data supporting the conclusions of this article will be made available by the authors on request.
